# A Rare Case of Acute Transverse Myelitis (ATM) and Acute Motor and Sensory Axonal Neuropathy (AMSAN) Overlap

**DOI:** 10.7759/cureus.5426

**Published:** 2019-08-19

**Authors:** Zarmina Javed, Waseem T Malik, Omair ul haq Lodhi

**Affiliations:** 1 Internal Medicine, Shifa International Hospital, Islamabad, PAK; 2 Neurology, Shifa International Hospital, Islamabad, PAK

**Keywords:** acute transverse myelitis, overlap syndrome, guillain barre syndrome

## Abstract

Concomitant acute transverse myelitis (ATM) and Guillain-Barre syndrome (GBS) is described as GBS and ATM overlap. Its presentation varies greatly, thus making the diagnosis difficult. Overlap syndrome is more commonly associated with acute motor axonal neuropathy (AMAN) subtype of GBS. However, we present a case of a middle-aged gentleman with combined ATM and acute motor and sensory axonal neuropathy (AMSAN) subtype of GBS. This combination is quite rare, and only a few cases have been reported so far.

## Introduction

Guillain-Barre syndrome (GBS) is an acute immune-mediated polyradiculoneuropathy of the peripheral nervous system which comprises of several subtypes, while acute transverse myelitis (ATM) is an immune-mediated disorder of the central nervous system (CNS) [1-2].

The occurrence of Guillain Barre syndrome (GBS) and acute transverse myelitis (ATM), either concurrently or sequentially, is defined as GBS and ATM overlap [3]. This overlap is quite rare and its diagnosis, challenging. Twenty three cases of overlap syndrome reported so far [4]. These cases were mostly preceded by gastrointestinal infections such as Campylobacter jejuni while other cases were associated with Zika virus, Mycoplasma pneumoniae, Legionella pneumophila, Bartonella henselae, Influenza virus, and Paramyxovirus [5-10]. Majority of them were reported in the pediatric population and were commonly associated with acute motor axonal neuropathy [11].

We present a rare case of concurrent acute motor and sensory axonal neuropathy (AMSAN) and acute transverse myelitis in a 34-year-old gentleman. 

## Case presentation

A 34-year-old gentleman, with no known comorbidities, presented with bilateral lower limb numbness and weakness for seven days and upper limb weakness for four days. He also reported three days of self-limiting diarrhea, which occurred ten days back. The patient had no respiratory symptoms or any history of recent vaccination. On examination, vitals were normal, and neurological examination demonstrated bilateral motor power of 3/5 in lower limbs and 4/5 in upper limbs, diminished reflexes, and bilateral down-going planters. Sensory examination revealed decreased tactile sensations in all limbs without any well-defined sensory level. Cranial nerves examination was normal, and the Glasgow Coma Scale (GCS) score was 15/15.

**Figure 1:**
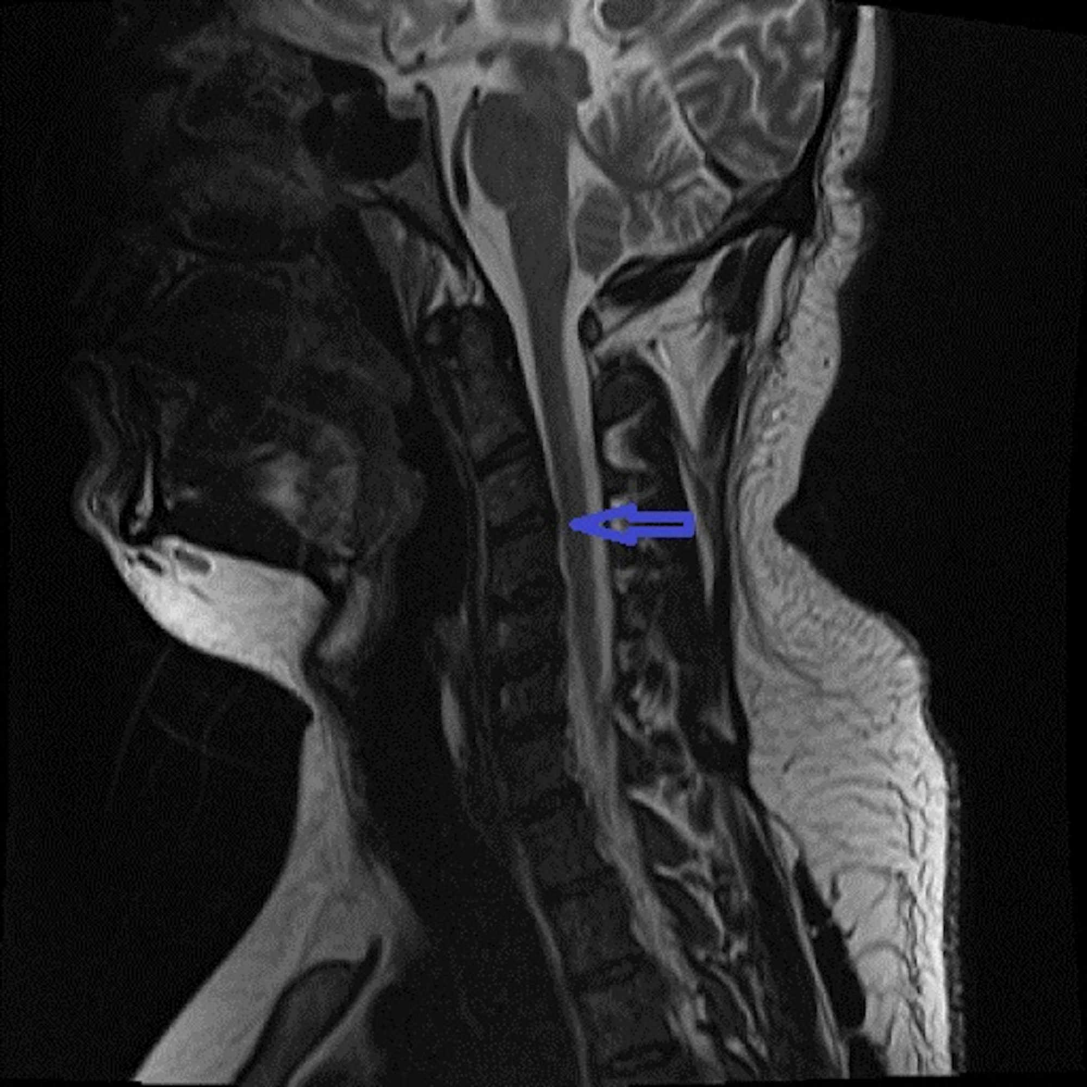
Magnetic Resonance Imaging (MRI) of the cervical spinal cord Sagittal T2- weighted MRI showed hyper-intense intra-medullary signal opposite C2-C3 through C4-C5 level (arrow).

**Figure 2:**
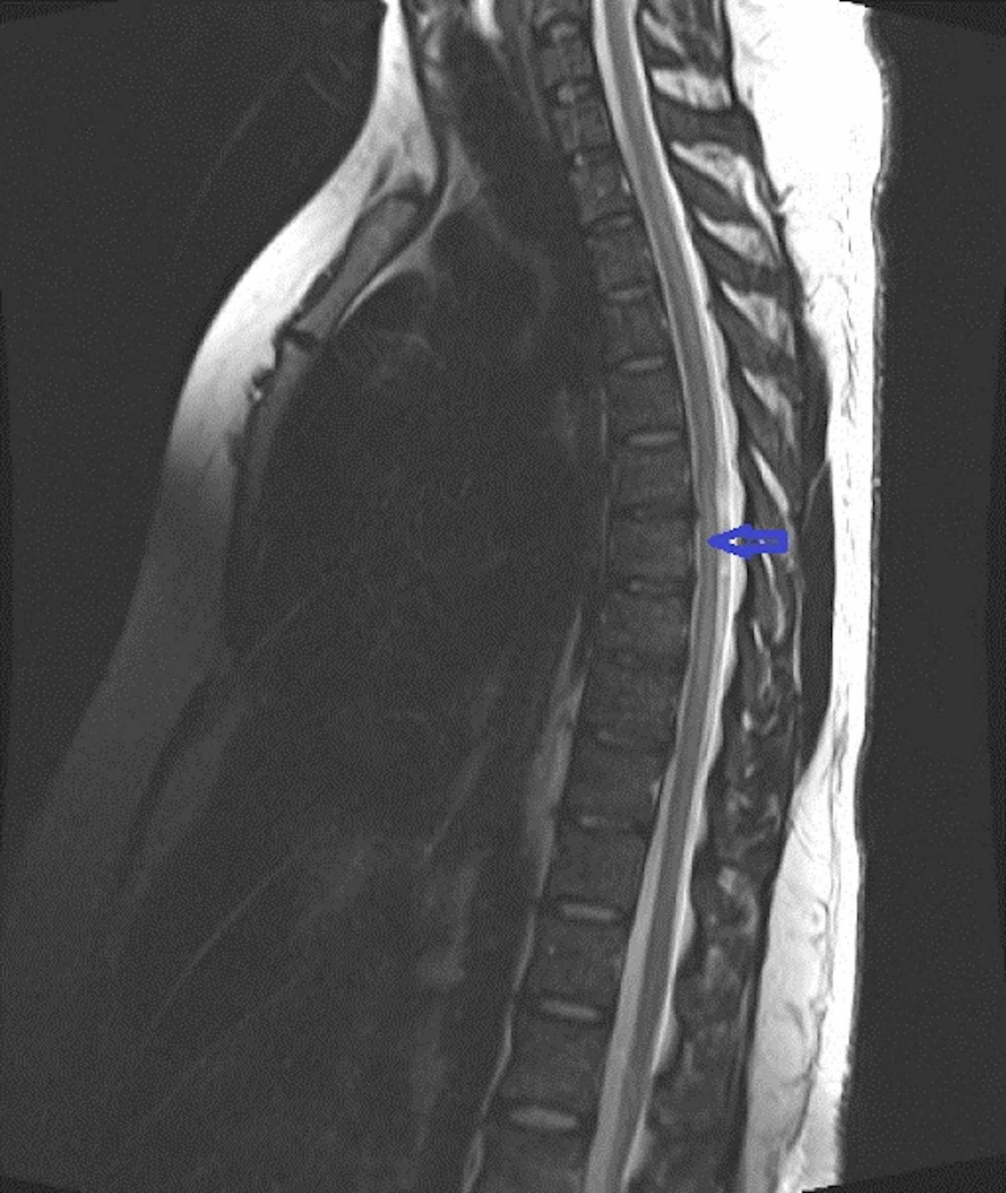
Magnetic Resonance Imaging (MRI) of thoracic spinal cord Sagittal T2- weighted MRI showed extra-medullary intradural hyper-intense signal in thoracic region (arrow).

The patient was admitted to the intensive care unit and observed closely for any respiratory depression or autonomic instability. A detailed workup for acute transverse myelitis was initiated while he was being managed with intravenous corticosteroids. The Magnetic Resonance Imaging (MRI) of the cervical spine without contrast was performed immediately, which showed T2 hyper-intense intra-medullary signal in cervical cord opposite C2-C3 through C4-C5 level. Another extra-medullary intradural T2 bright cerebrospinal fluid intensity area was noted at the T6 level (Figures 1, 2). Narrowing of the central canal and foramina was not observed on the MRI. Cerebrospinal fluid (CSF) analysis revealed normal white cell count, elevated proteins, and negative bacteriological testing. CSF polymerase chain reaction (PCR) was negative for Herpes simplex virus and Mycobacterium tuberculosis. Stool and bacterial cultures were all negative.

On the second day of admission, the patient’s clinical examination had worsened where lower limbs were found to be flaccid bilaterally with motor power of 2/5. The remaining examination was unchanged. Nerve conduction studies were ordered, which revealed predominant axonal motor and sensory neuropathy affecting lower limbs more than upper limbs (Table 1). The acute history and nerve conduction studies were suggestive of acute motor and sensory axonal neuropathy (AMSAN) variant of GBS. The treatment plan was switched from intravenous corticosteroids to intravenous immunoglobulin (IVIG) therapy (0.4g/kg/day for five days). Rest of the investigations, including GBS serology, serum anti-aquaporin-4 antibody, and rheumatologic workup, was negative.

**Table 1:** Nerve conduction study (including only the nerves involved in our patient) * Abnormal value

	Right side				Left side			
	Latency (ms)	Amplitude (mV)	Velocity (m/sec)	F wave latency (ms)	Latency (ms)	Amplitude (mV)	Velocity (m/sec)	F wave latency (ms)
Motor nerve conduction study								
Tibial nerve (ankle)	4.6	3.0*			4.9	2.4*		
Tibial nerve (knee)	16.1	1.1*	38	60.8*	15.7	1.1*	41	62.2*
Deep peroneal nerve (knee)					_	Not recordable	_	_
Deep peroneal nerve (above knee)					_	Not recordable	_	_
Deep peroneal nerve (below knee)					_	Not recordable	_	_
Sensory nerve conduction study								
Sural nerve	_	Not recordable	_		_	Not recordable	_	
Superficial peroneal nerve	_	Not recordable	_		_	Not recordable	_	

The patient improved gradually with IVIG therapy. After nine days of hospital stay, he was discharged home with 4/5 power in lower limbs, 5/5 power in upper limbs, +1 deep tendon reflexes and normal sensory examination.

## Discussion

The overlap of Guillain Barre syndrome (GBS) and acute transverse myelitis (ATM) is defined as the concurrent or sequential occurrence of GBS and ATM. This may be explained by the presence of a common epitope of myelin in the peripheral and central nervous system [3]. Overlap syndrome is generally considered to be rare and more commonly associated with acute motor axonal neuropathy (AMAN) subtype of GBS. However, we present a case of a middle-aged gentleman with concurrent acute motor and sensory axonal neuropathy (AMSAN) and ATM. During our literature review, we identified only three reports of similar overlap; a pediatric case, a 28-year-old woman, and a 64-year-old male [8,10-11].

The clinical presentation of GBS and ATM overlap varies extensively. One review broadly classified the clinical presentation of overlap syndrome into three categories: patients with positive pyramidal signs and areflexia or hyporeflexia, those who suffered pain at the onset of the disease and those with respiratory compromise requiring ventilator support. Sensory loss and incontinence indicate concurrent ATM [4]. In our case, the patient had motor weakness (more in the lower limbs), hyporeflexia, sensory loss, and extreme flaccidity of lower limbs, which worsened with corticosteroid therapy.

During the early stages of overlap syndrome, the diagnosis is challenging. Hence, most of the patients are initially diagnosed with GBS or ATM alone. Electrophysiological studies help with the diagnosis of GBS, while spinal cord MRIs are fundamental for the identification of ATM [4]. Our patient was initially diagnosed with ATM based on MRI findings. The very next day, due to the lack of clinical improvement with corticosteroids, nerve conduction studies were performed, which revealed AMSAN.

Currently, the first-line therapy for GBS and ATM overlap is not well defined [4]. Corticosteroid therapy and plasmapheresis are effective for ATM [12]. The first-line therapy for GBS is intravenous immunoglobulins (IVIG) or plasmapheresis while corticosteroids alone have not proven to be beneficial in the management of GBS and may worsen the weakness [5]. If patients of ATM do not show improvement with initial corticosteroid therapy, the next treatment options are plasmapheresis or IVIG [13]. Our patient with combined GBS and ATM showed worsening of symptoms with initial corticosteroid therapy. He was advised to discontinue corticosteroids and begin plasma exchange or IVIG treatment. The patient opted for IVIG therapy, which was given at a dose of 0.4g/kg/day for five days.

Combination therapy of corticosteroids and IVIG has not proven to have a beneficial outcome; a review study revealed that less than 50% of patients benefited from the combined therapy [4]. In our case, only IVIG therapy was given, and the patient improved, ultimately having an almost normal neurological examination.

## Conclusions

Overlap of ATM and GBS is rare and can often be missed due to varied clinical presentations. Hence, it should be considered in patients who have an acute progressive weakness with combined upper and lower motor neuron signs on examination. As explained in our case, except for corticosteroids on the first day, IVIG therapy was given, which improved the patient’s condition. However, further studies are required to identify the exact etiology and first-line therapy for overlap syndrome.
